# A Meta-analysis on the Effect of Expertise on Eye Movements during Music Reading

**DOI:** 10.16910/jemr.15.4.1

**Published:** 2022-07-28

**Authors:** Joris Perra, Alice Latimier, Bénédicte Poulin-Charronnat, Thierry Baccino, Véronique Drai-Zerbib

**Affiliations:** LEAD Université Bourgogne Franche-Comté, Dijon, France; CHART/LUTIN Université Paris 8, Paris, France; *The first two authors equally contributed to the first authorship

**Keywords:** Expertise, music reading, eye tracking, meta-analysis, fixation duration, number of fixations, saccade amplitude, gaze duration

## Abstract

The current meta-analysis was conducted on 12 studies comparing the eye movements of expert versus non-expert musicians and attempted to determine which eye movement measures are expertise dependent during music reading. The total dataset of 61 comparisons was divided into four subsets, each concerning one eye-movement variable (i.e., fixation duration, number of fixations, saccade amplitude, and gaze duration). We used a variance estimation method to aggregate the effect sizes. The results support the robust finding of reduced fixation duration in expert musicians (Subset 1, g = -0.72). Due to low statistical power because of limited effect sizes, the results on the number of fixations, saccade amplitude, and gaze duration were not reliable. We conducted meta-regression analyses to determine potential moderators of the effect of expertise on eye movements (i.e., definition of experimental groups, type of musical task performed, type of musical material used or tempo control). Moderator analyses did not yield any reliable results. The need for consistency in the experimental methodology is discussed.

## Introduction

### Expertise in music reading

Music reading is a demanding task that consists in extracting visual information from the score in order either to study it in preparation for a subsequent performance (i.e., silent reading) or to perform the music by playing an instrument or singing while discovering the score (i.e., sight reading). Since the seminal research of Jacobsen (48) and Weaver (105), researchers in the field of psychology of music have shown huge interest in how music-reading skills develop and the nature of the underlying mechanisms (55). Learning how to read music is part of the musical training in the Western classical music tradition. This skill has been shown to evolve greatly with experience up to the point at which real expertise is acquired.

In terms of cognitive mechanisms, Chaffin and Imreh (12), Williamon and Valentine (106), and Drai-Zerbib and Baccino (e.g., 18) used the *long-term working memory theory* developed by Ericsson and Kintsch (23) to provide a theoretical framework for the development of expert music reading. This theory proposed that “cognitive processes are viewed as sequence of stable states representing end products of processing. In skilled activities, acquired memory skills allow these end products to be stored in long-term memory and kept directly accessible by means of retrieval cues in short-term memory, as proposed by skilled memory theory” (23; p.211). This kind of expert memory can explain the differences in cognitive processes between expert and non-expert musicians. Expert musicians are able to rapidly focus on relevant information to process. Acquired memory skills also lead expert musicians to use better visual pattern recognition processes (103). This perceptual advantage enables them to rapidly access useful information, using visual features as larger patterns (6; 29; 61).

Music-reading expertise has been investigated with different methodological approaches based on psychophysical paradigms (12; 91), brain imaging techniques such as fMRI and EEG (e.g., 51; 67; 109) and eye tracking (12; 18; 20; 99; 103). The present review focuses on studies that investigated music-reading expertise with eye-tracking systems, a methodology commonly used in the visual expertise literature (75; 72; 88 for recent reviews).

This meta-analysis proposes to investigate usual metrics applied in eye-tracking research on music reading. Even if there are not so many studies in this field, this kind of analysis can represent a first maturation for the field of music-reading research that can help future research. Furthermore, the question of how eye movements evolve as a function of expertise in a given task is a key issue in various domains when studying the effect of expertise. This topic has been studied in domains such as chess (81), medicine (52; 87), sports (47) or video games (17). Sheridan et al. (88) emphasize that the study of eye movements in music reading could help to develop expert memory theories and their limits insofar as music reading is a specifically multimodal task, which offers a very unique angle of expertise research, especially to understand how expertise relies on handling multisensory information.

### Eye tracking in music reading

Since the findings of Yarbus (110), it has been acknowledged that there is a strong relationship between eye movements and underlying perceptual and cognitive processing (46; 49—eye-mind link hypothesis; 79). The main purpose of the visual system is to gather information in order to guide comprehension, decision-making, and motor planning. The investigation of visual patterns during the encoding of information from the environment opens a window onto the brain that makes it possible to study the underlying information processing mechanisms (33).

Although there are many ocular variables that are used in this field of research such as the number of blinks, pupil size or scanpath analysis, two main eye movements are usually extracted and analyzed: fixations (i.e., short pauses that focus on elements to process and information intake) and saccades (i.e., successions of alternating jumps from one fixation position to another). The information extracted from a fixation is integrated to provide a meaningful basis for further processing and guides the saccade that leads to the next fixation and so on (76).

Text reading has been extensively studied in eye-tracking paradigms (62, 63; 76). Eye-movement measures are particularly appropriate for studying music reading because the way the eyes scan the musical score determines the quality of information processing and the accuracy of the subsequent performance (24). Eye-movement investigation goes beyond “behavioral-only” experiments by generating objective and understandable indicators of 1) how musical scores are efficiently scanned by the eyes; 2) how different features can modulate the pattern of eye movements while reading music (e.g., aims, type of task, complexity of the musical structure, tempo); and 3) how reading music evolves with training by making the analysis of the score more efficient. Investigating these indicators helps provide robust insights related to the musical mind, and notably on how musicians integrate the scores as meaningful information so that the music is automatically interpreted and performed quickly and accurately just as text is by expert readers (2; see for a review 60).

The study of eye movements permits accurate temporal measures which, in turn, allow a precise description of the time course of early (e.g., first fixation duration) or late-occurring cognitive processes (e.g., second pass duration). The following sections describe the main variables used in the field of eye tracking in music and in what conditions these variables are modulated by musical expertise.

### Eye-movement variables

Multiple metrics derived from eye-position data can provide information about perceptual and cognitive processes during a particular activity (22; 46). Both fixations and saccades provide different information in the context of music reading and are modulated by several factors, notably the level of musical expertise (see the following section *Main results on eye movements modulated by expertise).* Music-reading research is usually focused on global metrics; that is, fixations or saccades are collected for entire stimuli or trials. However, a non-negligible number of studies also includes local visual metrics on a specific area of interest, within the stimuli (e.g., on each bar of a musical score).

The eye-hand span (EHS) is another metric reported in music-reading studies. This metric is defined as the distance that the eyes are ahead of the hand in playing (for a review, see 72). For the scope of the present meta-analysis, we did not include experiments investigating the effect of expertise on the EHS because this is assessed in the case of a sight-reading task only, and this literature is a particular one. Identifying such studies to conduct a meta-analysis means designing a different search protocol and defining different inclusion criteria.

It is important to note that depending on the eye-tracker device supplier, and on the research papers, the terms used to describe eye-movement measures suffer from a lack of standardization. This section gives general definitions of eye-movement variables rather than specific designations and descriptions. The huge variety of eye-tracking variables and their naming has been taken into account in the methods and analyses of the present meta-analysis.

### Fixation measures

Different measures of fixation durations are commonly used in music-reading studies. One of them is the time spent looking at specific parts of the musical scores. It reflects the time needed to process the information and is expressed in milliseconds (76; 78). Depending on the study, there is a distinction between the average fixation duration while discovering the score for the first time (e.g., first-pass fixation duration or visit duration) and the average “re-fixation” duration when the eyes read the same passage of the score for a second time (e.g., second-pass fixation duration or re-visit duration). There is also a distinction between the average fixation duration (i.e., how long the average fixation lasted for) and the total fixation duration (i.e., sum of the durations of all the fixations). Some research also reports the dwell time (i.e., time that gaze remains in a particular area on the stimuli, from entry to exit; 46) or the gaze duration, which represents the sum of all fixations made on an element prior to a saccade to another element (76).

During music reading (either silent or sight reading), fixations typically last between 350-400 ms on average (10; 36; 60; 103). This is longer than the average fixation duration observed during silent text reading (225 ms) or during oral text reading (275 ms; 76). Furthermore, the number of fixations on a specific part of the stimulus (i.e., an area of interest) is a complementary measure of the duration and provides an insight into the level of difficulty involved in decoding musical scores and determining the meaning of any given item of information in the stimulus (76). The number of fixations in an area of interest is usually correlated with the total dwell time (46).

### Saccade measures

Saccades are more related to attentional shifting between fixations, either controlled towards another note or group of notes or automatic towards an unpredictable piece of information such as a violation of the musical structure (59). As also found in the literature on text reading, there is usually a distinction between progressive and regressive saccades. Progressive saccades (also known as forward saccades, in the case of dextroversial writing, are left-to-right movements and link first-order fixations with each other during first reading. Regressive saccades (also known as backward saccades) are right-to-left movements occurring when the eyes move to a preceding location (e.g., to the beginning of the score or to the preceding note).

The number of progressive saccades might reflect initial processing during the discovery of the musical material. The number of regressive saccades might reflect delayed processing to retrieve more information, indicating additional control of the part of the score already read. A regressive saccade might also be a jump back at the end of musical phrases to check information retrieved earlier.

The saccade amplitude (or the length of the saccade) measures the ability to go from one part of the piece to another, and it is typically measured in degrees of visual angle. This measure is also related to variations in task demand in terms of the workload required by the current cognitive processes (107). The saccade amplitude is usually shorter in more difficult tasks, while it is larger when participants look at meaningful information (34; 73). However, one limitation of using saccade amplitude is its idiosyncratic nature (i.e., all participants have their own basic value for this indicator; 46).

Overall, the fixation and saccade metrics have been extensively investigated not only in music-reading studies, but more generally speaking in the expertise literature. These metrics can be used as determinants, markers of such expertise, because numerous findings provided evidence that these eye-movement metrics depend on the level of musical expertise.

### Eye movements in musical expertise

The eye movements of experts performing various domain-relevant tasks have been investigated in the eye-tracking literature (74; 81; 85; 88). Generally, depending on the goal, experts are better able to focus their gaze and attention on relevant and informative aspects of the stimulus than non-experts or novices. This is in line with the *information-reduction hypothesis* formalized by Haider and Frensch (39).

The chunking theory also suggests that experts have a perceptual advantage because they acquired domain-specific memory structures (i.e., chunks) during learning and extensive practice (13; 30; 31). Thus, applied to music, this theory postulates that experts process domain-specific stimuli as chunks (e.g., meaningful groups of notes such as chords or arpeggios) instead of as individual features (e.g., a single note).

Moreover, experts are also able to process information in parafoveal vision more easily (1). Indeed, the visual intake takes place not only in the foveal projection but also around it: this is referred to as the perceptual span. The perceptual span is the amount of visual information that is processed during a fixation (76). As in many fields, in music reading, larger perceptual span is a hallmark of expertise; and this may explain differences in the visual pattern, in accuracy, and in velocity for behavioral measures (88). Related to the chunking theory, the larger perceptual span of experts reflects their ability to process domain-specific visual features as larger chunks. This is why experts usually make longer saccades when performing domain-related tasks (80; 86).

Even if the number of studies on music reading is quite limited and with results that do not systematically converge, effects of expertise are also observed in music reading similarly to those found in text reading. The most robust result is that expert musicians show reduced fixation durations (21; 18, 19; 78; 103). Because fixation duration is predictive of processing time, longer fixations may indicate that encoding the musical stimuli imposes greater cognitive demands in non-expert musicians (32; 77). Thus, novices produce more and longer fixations, revealing the unsystematic reading of note combinations (i.e., they read music note by note), whereas experts generally produce fewer and shorter fixations because they exhibit a more systematic reading of scores involving the recognition of known musical patterns Waters et al., 104).

Overall, the replicated finding is consistent with the *long-term working memory theory* (23, which suggests that the retrieval structures related to musical knowledge in memory enhance the encoding of musical material and thus its subsequent retrieval. Furthermore, the fact that expert musicians differ from non-expert musicians on the number and duration of fixations suggests that the intake of information is enhanced in experts so that the search leads to rapid and accurate detection of relevant information.

Related to the visual expertise research, other theories that are not mutually exclusive also support this effect of expertise in music reading, such as the holistic processing model (53) and the global-focal search model (68). Finally, under certain experimental conditions, several studies have shown that the fixation duration in expert musicians can be disrupted when unexpected events are introduced in the score (i.e., melodic, harmonic, or rhythmic patterns that are not consistent with musical rules), while fixation patterns in non-experts are less modulated by such disruptions (5; 19; 69; 92).

Measures of saccades have received less attention than measures of fixations in studies on the effect of expertise in music reading. Studies have reported a reduction in the number of regressive saccades with increasing music-reading expertise, as observed in text reading. This result suggests that less expert musicians need to increase regressive fixations in order to re-check musical information during reading or likely due to a misunderstanding of the musical pieces (18; 69). However, Arthur et al. (5) did not find an effect of expertise in the proportion of either forward or regressive saccades during a music sight-reading task. This result is probably due to the unconventional score notation used in this study (i.e., unexpected disruptive spaces between notes). This way of writing music increases the saccadic latency and might have disturbed chunking mechanisms.

Moreover, saccade amplitude seems to be also sensitive to the musical expertise. Some studies found that expert musicians exhibited larger saccades when reading scores, which was consistent with findings in the field of text-reading activity (18; 35; 78; 103). The ability of expert musicians to gather groups of notes into a single unit for processing (i.e., chunking) rather than reading each note individually is very similar to the process used by expert-text readers (26; 91; 99; 108). Because saccades are limited in length by the perceptual span, which is shorter in non-expert musicians, this might also explain the effect of musical expertise on saccade amplitude (76). Recently, Maturi and Sheridan (61) showed that this theory of a larger perceptual span would also apply to expert musicians, who have different search strategies than non-experts. However, previous studies using a moving-window paradigm did not find any effect of musical expertise on the saccade amplitude (28; 99), which emphasizes some inconsistency in the literature.

Overall, the effect of musical expertise in music reading seems to involve focusing attention on the more relevant information, thus collecting less information as well as a faster processing of musical material because experts use retrieval structures, which result from musical knowledge learned from an intensive musical training (10; 19, 20; 69). Such an expertise might account for the fact that expert musicians adhere less closely to the information written in the musical score than non-experts (18). Furthermore, expertise in music reading seems to be characterized by a parafoveal processing advantage (88).

### Moderators of the effect of musical expertise in music reading

Depending on various features of the experimental set-up, the differences in eye-movement metrics as a function of music reading expertise vary from one study to another. More proficient musicians have been shown to make a similar number of shorter fixations (35), fewer fixations of similar durations (28) than less proficient musicians, while expert musicians have been shown to make a similar number of longer fixations (61), or show no differences in the number and duration of fixations compared to novices (5). This lack of consistency might be explained by methodological differences between studies (75).

First, eye-movement metrics vary depending on the music-reading task, and in particular if the reading activity also requires the participant to play the piece. Sight reading is a specific musical ability that requires the musician to produce the music with little or no prior experience of the piece to be played. By contrast, silent music reading is another way for musicians to study a musical piece or look for information and does not include a production phase even if this activity also involves sensorimotor processing (93). As found in the meta-analysis of Gegenfurtner et al. (27) on eye-tracking studies of expertise differences, the task characteristics modulate the size of expertise differences. Thus, the gaze behavior used for sight reading (performance tasks) and silent music reading (nonperformance tasks) are expected to be quite different and might also be modulated by musical expertise (21; 88).

Second, notational variants or disturbances (e.g., syntax violations) modulate the fixation pattern of musicians and interact with the level of musical expertise (2; 5; 20; 50). To extract the information required to play a melody, expert-music readers are not sensitive to the same parameters in the musical structure as non-experts.

Third, the methodological choices concerning whether to impose a faster or slower tempo or whether or not to impose a tempo could be a decisive moderator in measuring the evolution of eye movements as a function of expertise in a sight-reading task. On the one hand, tempo has an impact on the duration of the notes to be played and thus the time available to decipher them. In a study by Truitt et al (99), musicians were split into two skill groups based on their playing tempo. The musicians who played at the highest tempo were also those who had shorter fixation durations. On the other hand, imposing or not the tempo could induce different eye movement behavior. For example, in a study by Penttinen et al. (71), expert musicians had a greater gaze activity by inspecting more adjacent Areas Of Interest (AOIs) than less expert musicians in a tempo-controlled situation. These results could be explained by the fact that expert musicians process musical information faster than less expert ones and use the remaining time available between two beats to explore the score. In the case of no tempo control, musicians who decipher visual information more quickly will be more likely to play the score faster (99) rather than to use the free time between beats to explore the score. For that reason, controlling the tempo could moderate the effects of expertise on eye movements.

Other parameters which can modulate the effect of expertise, are the variability in the way musical expertise is measured (based on the position in the institution or on the number of years of instrumental practice), and the level of sight reading itself (based on the playing performance of a specific musical piece or on the scores achieved in a sight-reading test). The criteria used to assign participating musicians to the expert or non-expert groups can vary across studies because there is no single way to conceptualize music expertise. The “difference” between two groups with a different level of musical expertise is not always comparable across studies and this can explain some inconsistencies. For example, some studies compare groups of expert musicians with non-experts (i.e., a population of musicians from different expertise levels, 19; 69) while others compare experts with a population of novices (i.e., non-musicians, 104; 103).

Finally, the way analyses of eye-movement measures are conducted might also modulate the potential difference between expert and non-expert musicians, notably because the features of eye-tracking devices can vary (e.g., sampling frequency, accuracy, resolution, fixation detection algorithms). Furthermore, researchers have used different methods to clean and process the eye-movement data (e.g., minimum values for the duration of a fixation) and the definition of eye-movement parameters varies considerably at the level of semantics, with several different terms sometimes being used to name one and the same eye-movement measure.

### The present study

Meta-analysis allows the conversion of various studies on the same topic into one single quantitative review. The compilation of different results relating to the same effect makes it possible to portray what is called the “true effect”, reflected by the computation of the pooled standardized effect size (7). This method lends robustness to results, which appear to have been highly replicated in the literature, or may reveal an overall null effect of a variable, which the community has believed to be significant (4; 90). Furthermore, meta-analysis makes it possible to investigate different study-level variables, and this might account for equivocal results (i.e., moderator analysis), and can show how these variables may modulate the size of a specific effect, thereby permitting the interpretation of variability observed across studies. Finally, meta-analysis also may shed light on publication bias issues (83).

To our knowledge, no meta-analysis has been published on the effect of musical expertise on music reading through eye movements. In 2014, Mishra focused on the sight-reading literature and published two meta-analyses (64, 65). One of these investigated the relationship between various stable cognitive characteristics (e.g., IQ and personality) and sight-reading abilities (i.e., correlation between sight-reading performance and other continuous measures). Her results showed that factors that can be improved with practice, such as music-reading activities, correlated more strongly with sight-reading abilities than did stable cognitive characteristics. The other meta-analysis (65) investigated the benefits of various interventions in enhancing sight-reading abilities and found that training eye movements through controlled music reading can improve sight reading.

Overall, these findings are in line with the general idea that eye movements evolve with expertise. However, the analyzed research did not include eye-tracking studies. We thus identified a need for a meta-analysis on eye tracking in music reading, a field of research that has contributed to understand music cognition for decades and that is still growing with the development of new methodologies. The challenge in this research area is the limited number of studies to provide reliable and relevant results. However, meta-analyses frequently include only a small number of studies, as revealed by a review of the Cochrane Library (i.e., half of the meta-analyses reported in the Cochrane Library concerned two or three studies, 100). Moreover, even applied on few studies, meta-analyses can provide a basis with very helpful results to subsequently carried out larger meta-analyses (i.e., Wang et al., 2007 examined media effect on performance including 11 studies; Kingston, 2008 included 16 studies; ten years later Delgado et al., 16 included 38 studies).

A major problem in research synthesis is that studies usually differ in their methodology, data collection, and analyses. Indeed, there may be a lack of consistency in the methodology used for eye-movement research during music reading (56; 72; 75). This leads to a non-negligible variability across studies, which might account for inconclusive data in some cases. Moreover, the time lag between publications on eye tracking in music reading is large, with the result that the paradigms used have varied as eye-tracking devices have evolved (from 1943 to 2020), and this makes it more difficult to integrate the main findings into a coherent whole. Finally, as set out in the preceding section, several factors might modulate the effect of musical expertise in music reading and be responsible for the lack of any clear conclusion concerning the eye-movement indicators of musical expertise, especially in the case of the saccade measures.

For the present study, we formulated the following main question: how does musical expertise modulate eye movements when reading a musical score? To do so, we focused on eye-tracking research in the domain of music reading. We aimed at focusing on the most relevant eye-movements metrics, which are related to the perceptual-cognitive processing in music reading and for which the literature demonstrated differences between experts and non-experts in other domains (i.e., durations and number of fixations, saccade amplitude, dwell time or gaze duration; 9; 27). The second aim was to investigate how methodological factors might account for the differences of eye movements of expert and non-expert musicians (i.e., the type of reading task, the type of musical stimuli, the criteria used to assess the level of expertise, and the type of dependent eye-tracking variables used to investigate the effect of musical expertise).

Overall, this meta-analysis aimed at clarifying the direction of the results and revealing the amplitude of the potential effect of expertise in music reading. The present meta-analysis provides a first cumulative contribution to the field that could be enriched in the future. We also aimed at proposing suggestions for further research to provide a more comprehensive understanding of the perceptive and cognitive features of musical expertise. This work contributes to the general area of research on expert perception.

## Methods

### Search protocol

The following groups of keywords that we extracted from our research question were used in the relevant databases, namely *Web Of Science*, *PsychInfo*, *Scopus*: “Musicians eye tracking”, “Expert musicians eye tracking”, “Music reading expertise eye tracking”, “Expert music reading eye tracking”, “Eye-movement musicians”, and “Ocular patterns musicians”. These search criteria generated between 0 to 19 references depending on the database. We decided not to use Google Scholar because this generated too many references (i.e., between 5,870 and 87,900 references), most of which were not relevant (or simply duplications of those found in the other databases), given that this field of research is somewhat limited. The references in the identified studies were used to identify additional research and we also checked in specific databases for theses and dissertations (HAL and OATD). We also scanned references from recent reviews and published papers on the topic (38; 61; 72; 75) as well as articles citing the seminal paper of Weaver (105). Overall, our search protocol generated a total of 221 references. In situations where a dissertation led to the publication of a subsequent article, the two reports were considered as a single reference, and we screened the published reference.

### Inclusion Criteria

The present study focused on music-reading tasks: either reading at first sight leading to a playing/singing performance or silent music reading without any playing/singing performance (69). In line with our research question, a first dual screening was applied based on titles. This step generated 53 references (after removing duplicates). Then a second dual screening was applied based on abstracts of each reference in accordance with the following inclusion criteria: i) the research should be empirical (i.e., exclusion of review papers), ii) the research should explicitly contrast a group of expert musicians with a group of non-expert musicians, whatever the criteria used to assess the level of musical expertise, and iii) the methodology should include eye-movement measurements (i.e., eye tracking set-up). When the latter two criteria were not clearly stated in the abstract, we decided to include the reference for the next screening step to avoid excluding potentially relevant references.

After the dual coding of the abstracts, an inter-rater reliability was computed to assess the quality and consistency of the inclusion criteria. Reliability was high (%_agree_ = 96.2; Cohen’s kappa = 0.92), and discrepancies were resolved through discussion. This second screening led to 32 references. We retrieved the full text papers for each of the 32 references, and when this was not possible, for example because the reference was a conference communication, we contacted the authors (three references were concerned, only the data from the study by Lörch, 58 could be included using this method).

The full-text screening step led to the list of eligible references to be included in the final analyses. We applied new inclusion criteria. Firstly, the study had to include a clear music-reading task involving the reading of musical scores even if other behavioral tasks were included in the procedure (e.g., performance accuracy, memory, or motor tasks). Secondly, the effect of musical expertise had to be investigated using a between-subjects design with two (or more than two) groups of different levels of musical expertise. We required a clear description of what was considered to be an expert and a non-expert participant, as well as of the criteria used to assess the type of musical experience. We did not include within-subject protocols investigating musical training effects (i.e., pretest versus posttest).

Thirdly, although we did not apply any criteria relating to the type of population, most of the studies included adult participants. Finally, the study had to report eye-movement data collected during the music-reading task as well as the types of eye-movement measures and the recording device. Last but not least, because the parameters could vary from one study to another, the types of musical stimuli had to be clearly reported. This step generated 16 eligible studies, leading to a total of 100 comparisons of eye-movement measures between expert and non-expert musicians.

**Figure 1.**
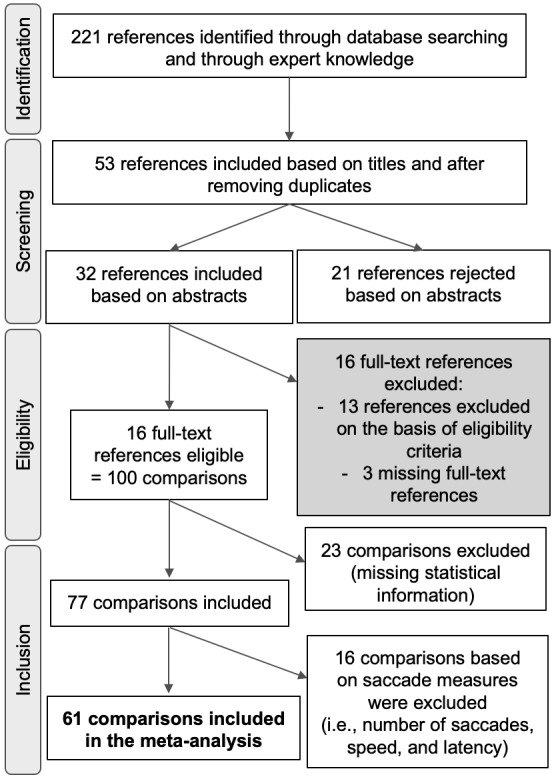
PRISMA group ﬂow diagram depicting study inclusion criteria. For each stage, we provide the number of included and excluded references, and the number of comparisons generated by the references.

The crucial criterion for eligibility was the availability of statistical data for computing effect size between different groups of musical expertise (and the standard error, *SE*). Some studies did not report the basic statistical information necessary to calculate effect sizes (e.g., descriptive statistics, *t* or *F* values). To overcome this issue, we could either ask the authors to provide descriptive statistics or compute them from the available raw data or included figures in order to extract means and *SE*s from graphs digitized using the *WebPlotDigitizer* software (https://automeris.io/WebPlotDigitizer/). This allowed us to recover missing relevant statistical information for each condition (e.g., means, standard deviations, *SDs,* and *SEs*). Following this last crucial inclusion guideline, 23 comparisons were rejected (from 6 references) while 77 comparisons satisfied all criteria for the final analyses. In these 77 comparisons, we identified several relevant eye-movement-dependent variables, which likely underlie different levels of processing for the musical stimuli and might reveal different effects of musical expertise. We therefore conducted separate effect size analyses based on these 77 comparisons. We decided to exclude ten comparisons based on the number of saccades (from three references). The number of saccades is rarely reported in music-reading studies because this metric might be less relevant to investigate the perceptual and cognitive processes in musicians. Moreover, two comparisons based on saccade latency and four comparisons based on saccade speed were excluded because of the very low number of comparisons and the low level of diversity in the subsets (coming from the same study, Arthur et al., 2016). Thus, a total of 61 comparisons (from 12 references) were separated into four subsets depending on the eye-movement variable: i) fixation duration (Subset 1: 29 comparisons), ii) number of fixations (Subset 2: 13 comparisons), iii) saccade amplitude (Subset 3: 8 comparisons), and iv) gaze duration in response to musical stimuli (Subset 4: 11 comparisons). Further details on each of these four subsets are presented in the *Results* section (see General study characteristics). Figure 1 summarizes the different screening steps, as proposed by PRISMA recommendations (66).

### Coding procedure and potential moderators

Several relevant items of information were extracted from each included reference both for descriptive purposes and also to assess methodological quality. These items were considered to be moderating variables in a second coding process conducted to determine the potential source of any heterogeneity in the different subsets of comparisons. The coded descriptive information is summarized in Table 1. We also coded any type of statistical information to compute effect sizes for final analyses (see Analyses). If one of these items of information was not included in the paper for a particular variable, it was coded as “Not Reported” (NR).

**Table 1.**
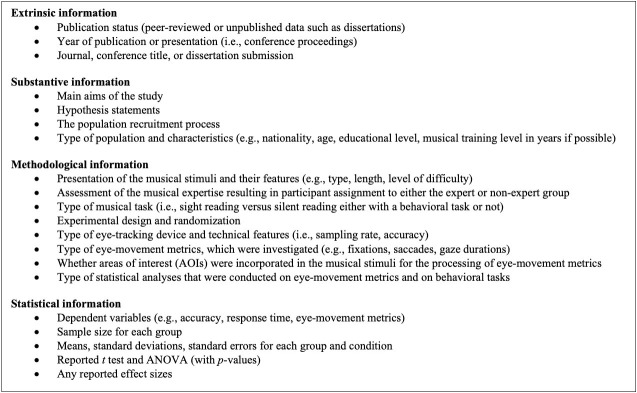
Summary of the coded information extracted from each included study

### Analyses

#### Computation of weighted mean effect size

We replicated the same methods of analyses as those proposed in Latimier et al. (54). Each subset of comparisons was analyzed separately. Effect size estimates were synthesized using Robust Variance Estimation (RVE)^1^ methods implemented in the R software (package: *robumeta,* 25).

This method makes it necessary to specify the correlation between within-study effects. We set the correlation between effect sizes at *ρ* = .80 (value given by default) and then conducted a sensitivity analysis to determine the impact of using alternative values between *ρ* = 0 and *ρ* = 1 (96). RVE uses the method-of-moment estimator to estimate between-study heterogeneity. This estimator, and the associated degrees of freedom, were adjusted for small sample sizes as recommended by Tipton and Pustejovsky (98). Results from RVE with these small sample corrections are likely to be biased (i.e., increased type I error rates) when the adjusted degrees of freedom are smaller than 4. Given the relatively small number of included samples, small-sample adjustments for hypothesis tests and confidence intervals (*CI*s; 98) were used for our analyses.

Three thresholds which are commonly used in psychological research are used to interpret the standardized mean difference (either for Cohen’s *d* or Hedges’ *g* with a value of 0.20 suggesting a small effect, 0.50 suggesting a medium effect, and 0.80 suggesting a large effect; 15).

#### Heterogeneity and publication bias assessment

Because we synthesize the effects of different studies into one single effect, it is important to assess the extent to which effect sizes vary within each meta-analysis: this is called heterogeneity. We thus report the magnitude of the heterogeneity *I²* (in %)*,* which represents the amount of variability not caused by sampling error (45). This indicator proposes three thresholds of interpretation, with *I*^2^ = 25% suggesting low heterogeneity, *I*^2^ = 50% suggesting moderate heterogeneity, and *I*^2^ = 75% suggesting substantial heterogeneity. Otherwise, *I²* is sensitive to the precision of the included studies (8).

We also report the estimated between-study heterogeneity (*T²*) for all analyses. *T²* is an estimate of the variance in the true effect sizes and is expressed in the same metric as the effect size (7). To determine the source of any heterogeneity, meta-regression analyses were performed for each subset separately. Following recommendations from Borenstein et al. (7), a minimum of six effect sizes for a particular moderator category was necessary for the moderator analyses to be appropriate.

Because some studies with negative or nonsignificant findings might not have been published and therefore were not included in this meta-analysis, the weighted mean effect size may be overestimated. We therefore estimated publication bias for each subset 1) with an inspection of funnel plot asymmetry and 2) by using Egger’s regression test. Funnel plots and Egger's regression are complementary methods used to determine whether there is a publication bias for each subset. At first, visual inspection of the funnel plot gives a clue about the level of asymmetry of the subset, namely to what degree we can expect a publications bias. If the funnel plot is asymmetrical, small studies with very high effect sizes should be considerably over-represented. Then, the Egger's regression test is a commonly used quantitative method that aims to confirm such asymmetry. When the funnel plot is symmetrical, emphasizing the fact that there is no publication bias, the Egger’s regression test should be significant, and the expected z-score should be scattered around zero.

## Results

### Subset 1: Fixation duration

Based on the fixation duration on the musical stimuli, we identified 10 studies dated from 1994 to 2019 involving a total of 29 effect sizes. Between two and four effect sizes were computed per study and fixation duration was expressed in milliseconds. The assessment of this measure varied between studies.

The measure of the average fixation duration was applied either for each AOI (i.e., bars, staves) or for the overall stimulus (i.e., scores). Other comparisons focused on first-fixation duration and second-fixation duration either as the total or the average duration (*k* = 14; with *k* standing for the number of effect sizes). Eighteen comparisons concerned a music-reading task without playing performance (e.g., silent reading and another task such as violation detection), whereas *k* = 11 comparisons concerned a music-reading task that involved a playing/singing performance (either true first sight-reading or rehearsed sight-reading). One study from which four effect sizes were computed did manipulate the type of musical task as an independent variable (21). Furthermore, the great majority of the included effect sizes were negative (*k* = 27), showing that the expert group in each of the comparisons tended to have shorter fixation durations in a music-reading task than the non-expert group. Only two effect sizes were very close to zero (5, ES2; 103, ES2).

Finally, it is important to note that two studies (*k* = 6) included three different groups of musicians: one with a high level of musical practice, one with a low level of musical practice or novices, and one with an intermediate level of musical practice (70; 104).

### Subset 2: Number of fixations

Based on this eye-tracking variable, we identified seven studies dated from 1994 to 2019 involving a total of 13 effect sizes. Between one and three effect sizes were computed per study and this measure was expressed as the count or rate. Most of these studies were also included in Subset 1 because fixation duration and number of fixations are complementary values. It is worth noting that three studies manipulated the type of musical stimuli in their experimental design by changing the features or structure of the stimuli (5; 18) or by manipulating the congruency between the auditory and the visual versions of each stimulus (19). Finally, and interestingly, *k* = 8 effect sizes were negative, showing that the expert group tended to produce fewer fixations during the music-reading task than the non-expert group; while *k* = 5 were positive and showed the opposite pattern of results.

### Subset 3: Saccade amplitude

Based on the saccade amplitude, we identified four studies dated from 1994 to 2013 involving a total of only eight effect sizes with between one and two effect sizes extracted per study. Researchers have largely neglected this specific eye-movement measure. Three of the effect sizes were close to zero, including the progressive saccade measures performed by Goolsby (35, ES1) and Drai-Zerbib and Baccino (18, ES1) and the measure comparing the saccade amplitude of less experienced readers with that of novices in Penttinen et al. (70). Otherwise, one was negative (35; ES2 on regressive saccades) while the other four were positive, showing that expert musicians can have larger saccades than non-experts.

### Subset 4: Gaze duration

We identified three studies, dated from 1994 to 2019, which investigated gaze duration, also called dwell time (20) and consists of the total gaze duration (in ms) on the stimulus or inside an AOI, including re-visits. This subset involved a total of 11 effect sizes, with three or four effect sizes computed per study. The three studies used a silent reading task that involved a measure of accuracy on a behavioral task (pair-matching, judgment matching, or modified note detection). Apart from one positive effect size (ES3 in Silva & Castro, 89), all were negative, a finding which is consistent with Subset 1: expert musicians have shorter gaze durations than non-experts when reading music.

**Table 2.** Descriptive information for the 12 studies included in the final analyses, across the four subsets. Panel A summarizes the parameters relating to the population, type of task and type of musical stimuli, while Panel B summarizes the parameters relating to the eye-tracking devices and eye-movement measures in each study.

**Panel A**								
**Included Studies**	***N*** ***total***	**Population**	**Expertise assessment**	**# of years of musical practice**	**Music reading task**	**Stimuli**	**Length of stimuli**	**Imposed tempo**
Goolsby (35)	24	Graduate students at a major university school of music 12 high-skilled readers versus 12 low-skilled readers	Based on the score obtained on the Belwin-Mills Singing Achievement Test: those with the 12 highest versus 12 lowest scores	NR	Rehearsed sight-reading	Four single-line written melodies selected from *Solfège des Solfèges* (classical treble clef, C major, and in 4/4 meter)	4 staves	Yes
Waters et al. (104, Experiment 2)	24	Group 1: 8 full-time music students from the Department of Music (University of Durham) Group 2: 8 musicians, students in psychology Group 3: 8 nonmusicians	Based on the score obtained on the Associated Board Grade examination. High-level musicians had passed an Associated Board Grade VIII examination while low-level musicians had all passed an Associated Board Grade IV, V, VI, or VII examination. The nonmusicians had little musical experience	NR	Silent reading	Sixty written melodies composed for the experiment. Each melody contained 5 to 8 different pitches and 3 to 5 values of different durations (written in 3/4 or 4/4)	2 bars of 5 notes	No
Waters & Underwood (103)	22	11 expert participants who had all achieved a high standard in at least one musical instrument associated with the treble clef register 11 participants, who were included in the novice group and were all at least partially familiar with musical notation	Based on the number of years of musical training (the novices all knew the names of the notes)	Experts having more than ten years of formal musical training versus non-experts having less than two years of musical training	Silent reading	Twenty melodies were written and consisted of simple scales or arpeggio structures in the treble clef. Four different types of stimuli: tonally and visually simple, tonally simple but visually complex, tonally complex and visually simple, tonally and visually complex	4 notes	No

**Included Studies**	***N*** ***total***	**Population**	**Expertise assessment**	**# of years of musical practice**	**Music reading task**	**Stimuli**	**Length of stimuli**	**Imposed tempo**
Drai-Zerbib & Baccino (18)	27	Piano students or teachers at the National Conservatory of Music in Nice, France 20 experts versus 7 non-experts	Based on music reading abilities, depending on the number of fixations across all musical stimuli (k-means method of classification)	Experts having at least 12 years of practice at the National Conservatory of Music versus non-experts having at least 6 years of practice	Rehearsed sight-reading	Sixteen two-stave partitions in treble clef were selected from Czerny, Bartok, Scarlatti. For each score, there was a written and an auditory version, as well as a version with phrase marks and one without phrase marks	4 bars	No
Drai-Zerbib et al. (21)	25	Piano students or teachers at the National Conservatory of Music in Nice, France 15 experts versus 10 non-experts	Based on their position in the musical institution	Experts having more than 12 years of practice versus non-experts having studied at the conservatory for six to eight years	Rehearsed sight-reading versus reading alone	36 piano excerpts in treble clef were taken from the classical tonal repertoire. Three versions of each excerpt were generated according to whether the fingering was given (difficult versus easy) or not	4 bars	No
Penttinen et al. (70)	37	Second-year education majors studying in a Finnish university to become primary-school teachers and taking part in a compulsory music training period lasting for one academic year 10 more experienced versus 11 less experienced versus 16 novices	Based on written reports on musical experience and ability (i) to read musical notation and (ii) to perform music from notation, as well as on the ability to perform music from notation in a simple sight-reading task at the start of the first measurement session (“Mary Had a Little Lamb” melody)	NR	Silent reading	A written melody from the Russian folk song repertoire called “Punasaappaat”	25 bars	Yes

**Included Studies**	***N*** ***total***	**Population**	**Expertise assessment**	**# of years of musical practice**	**Music reading task**	**Stimuli**	**Length of stimuli**	**Imposed tempo**
Drai-Zerbib & Baccino (19)	64	Music students or teachers at the National Conservatory of Music in Nice, France 26 experts versus 38 non-experts	Based on their position in the musical institution	Experts having more than 12 years of academic musical practice versus non-experts having from five to eight years of practice	Silent reading	48 excerpts in the treble clef were taken from the classical tonal repertoire for both visual and auditory presentations. An accent mark placed on one specific note and contributing to the prosody of the musical phrase (congruent versus incongruent position)	4 bars	No
Penttinen et al. (71)	38	The 14 experts were students of music performance at a Finnish arts academy or conservatory. The 24 non-expert musicians were education majors minoring in music education at the Department of Teacher Education of a Finnish university	Admission to both study programs necessitates passing program-specific tests of musicality and musical performance	The performance majors reported playing the piano for 14.8 years on average (*SD* = 5.2) while the education majors reported playing the piano for 11.5 years (*SD* = 6.5).	Rehearsed sight-reading	The original written melody and two slightly altered versions of the well-known children's song “Mary Had a Little Lamb” (key of C major)	8 bars	Yes
Arthur et al. (5)	20	University student body, participants self-selected 9 participants were assigned to the expert sight-reader group versus 13 to the non-expert sight-reader group	Based on the ability to play a short musical excerpt. An expert music sight-reader was defined as being able to perfectly or near perfectly perform a 6th Grade sight-reading examination piano piece set by the Australian Music Examinations Board	NR	True first-sight reading	Ten written melodies were composed in the treble clef, to be played by the right hand and within an octave span (normal versus disrupted)	4 bars	No

**Included Studies**	***N*** ***total***	**Population**	**Expertise assessment**	**# of years of musical practice**	**Music reading task**	**Stimuli**	**Length of stimuli**	**Imposed tempo**
Drai-Zerbib & Baccino (20)	53	Music students or teachers at the National Conservatory of Music in Nice, France 26 experts versus 27 non-experts	Based on their position in the musical institution	Experts having more than 12 years of academic musical practice versus the non-experts having between five and eight years of practice	Silent reading	20 single-staff excerpts of classical music in the treble clef. The 20 melodies of various levels of difficulty consisted of 18 to 58 notes written in various time signatures (2/4, 3/4, 4/4, 6/8) and presented in a tempo from 60 to 120 bpm	8 bars	No
Silva & Castro (89)	29	16 experts, who were musicians, music teachers or music students versus 13 non-experts (amateurs), who were not professionally involved in music	Based on the number of years of music reading training	Experts had been reading music for 21 years on average (*SD* = 7.8) versus non-experts, who had read music for 10 years on average (*SD* = 4)	Silent reading	48 written and auditory rhythmic sequences (time signature 2/4)	4 bars	Yes
Lörch (58)	149	75 expert music students versus 74 non-expert musically literate students	Based on the score obtained on the Gold Musical Sophistication Index. Music students had a Gold-MSI of 84.61 while the non-experts had a Gold-MSI of 68.88	The experts had nearly 10 years of practice while the non-experts had four to five years of practice	True first-sight reading	12 one-staff written melodies were composed with pitches randomly drawn from a set of five consecutive pitches. The rhythm was created by randomly combining four different bar types, each containing one type of note pair: eighth-eighth, quarter-quarter, eighth-quarter and quarter-eighth	4 bars	Yes

**Panel B**					
**Included Studies**	**Other behavioral tasks with accuracy measure**	**Eye-movement tracking equipment**	**AOI for eye-movement analyses**	**Types of eye-movement variable**	**# of computed Effect Sizes (ES) included in the final analyses**
Goolsby (35)	No	A Stanford Research Institute Dual Purkinje Image Eye-tracker (SRI), 1-ms sample of the location of the eyes (1000 Hz)	No	# of progressive fixations progressive fixation duration (in ms) progressive saccade amplitude # of regressive fixations regressive fixation duration (in ms) regressive saccade amplitude	6
Waters et al. (104)	Yes: pair-matching	A binocular infrared system with the Skalar IRIS system (Reulen et al., 1988). resolution = 0.10, sampling frequency = every 5 ms (200 Hz)	No	mean viewing time (in ms) # of fixations fixation duration (in ms)	9
Waters & Underwood (103)	Yes: pair-matching	An infrared beam from the cornea onto a photoelectric matrix (Wilkinson 1979). Accuracy of around ± 1character space, sampling frequency = every 4 ms (250 Hz)	No	# of fixations duration of the initial fixation prior to the first saccade (in ms) fixation duration (in ms) saccade amplitude	4
Drai-Zerbib & Baccino (18)	No	Eye-Gaze device (LC technologies : Fairfax). Sampling frequency = 60 Hz	Yes: nine (one on the clef, four on each bar of the right-hand staff and four on each bar of the left-hand staff)	# of fixations fixation duration (in ms) # of progressive fixations progressive fixation duration (in ms) # of regressive fixations regressive fixation duration (in ms) progressive saccade amplitude (in pixels) regressive saccade amplitude (in pixels)	4 (but 2 were defined as outliers in Subset 2)
Drai-Zerbib et al. (21)	No	Tobii Technology 1750TM eye-tracking system (Stockholm, Sweden). Sampling frequency = 50 Hz	Yes: nine (one on the clef, four on each bar of the right-hand staff, and four on each bar of the left-hand staff)	first-pass fixation duration (in ms) second-pass fixation duration (in ms) probability of re-fixation	4

**Included Studies**	**Other behavioral tasks with accuracy measure**	**Eye-movement tracking equipment**	**AOI for eye-movement analyses**	**Types of eye-movement variable**	**# of computed Effect Sizes (ES) included in the final analyses**
Penttinen et al. (70)	No	Tobii Technology 1750TM eye-tracking system (Stockholm, Sweden). Sampling frequency = 50 Hz, with a spatial accuracy of 0.5 degrees	No	absolute fixation duration (in ms) saccade amplitude (in pixels) proportion of linear saccades	9
Drai-Zerbib & Baccino (19)	Yes: modified note detection	Tobii Technology 1750TM eye-tracking system (Stockholm, Sweden). Sampling frequency = 50 Hz	Yes: four corresponding to each bar on the staff	first-pass fixation duration (in ms) second-pass fixation duration (in ms) # of fixations	6 (but 2 were defined as outliers in Subset 2)
Penttinen et al. (71)	No	Tobii TX300 eye-tracker manufactured by Tobii Technology AB (Stockholm, Sweden). Sampling frequency = 300 Hz	Yes, for the eye-hand span analyses: 12 corresponding to a quarter-note beat	fixation duration (in ms)	3
Arthur et al. (5)	No	Arrington Research ‘ViewPoint’ USB220 eye tracker. Sampling frequency = 220 Hz	No	# of fixations fixation duration (in s) fixation duration minus saccadic latency saccadic latency # of forward saccades forward saccade speed # of regressive saccades regressive saccade speed	8
Drai-Zerbib & Baccino (20)	Yes: modified note detection	SMI RED 500™ eye-tracking system. Sampling frequency = 500 Hz	Yes: nine at the global level (key signature and on bars 1 to 8), and three on bars with the modified note	first fixation duration (in ms) dwell time (i.e., gaze duration) # of fixations	8
Silva & Castro (89)	Yes: matching judgement between the audio and the visual musical stimuli	SMI RED eye-tracking system Sampling frequency = 120 Hz	Yes: four around each bar in the musical sequence	duration of the first fixation (in ms) first-pass gaze duration (in ms) total gaze duration (in ms)	4
Lörch (58)	Yes: span task	Tobii TX300 eye-tracker manufactured by Tobii Technology AB (Stockholm, Sweden). Sampling frequency = 300 Hz	No	# of fixations	1

**Table 3.** Computed effect sizes (in Hedges’g) for each included comparison (i.e., expert versus non-expert musicians) and for the four subsets depending on the eye-movement variables

**Comparisons of each subset**	**# of Effect Sizes (ES)**	**Effect sizes (*g*) Expert versus non-expert musicians**
**Subset 1: Fixation duration (in ms or s)**		
Goolsby (35)	ES1 (average duration) ES2 (average duration)	-0.92 -0.42
Waters et al. (104)^2^	ES1 (average duration) ES2 (average duration) ES3 (average duration)	-0.61 -1.55 -0.94
Waters & Underwood (103)	ES1 (average First-Fixation duration) ES2 (average Second-Fixation duration)	-1.12 -0.042
Drai-Zerbib & Baccino (18)	ES1 (average duration) ES2 (average duration)	0.38 -0.34
Drai-Zerbib et al. (21)	ES1 (average First-Pass Fixation duration) ES2 (average First-Pass Fixation duration) ES3 (average Second-Pass Fixation duration) ES4 (average Second-Pass Fixation duration)	-2.05 -1.86 -3.85 (o) -2.50
Penttinen et al. (70)^3^	ES1 (average duration) ES2 (average duration) ES3 (average duration)	-0.12 -0.60 -0.51
Drai-Zerbib & Baccino (19)	ES1 (total First-Pass Fixation duration) ES2 (total First-Pass Fixation duration) ES3 (total Second-Pass Fixation duration) ES4 (total Second-Pass Fixation duration)	-10.96 (o) -11.93 (o) -3.67 (o) -3.77 (o)
Penttinen et al. (71)	ES1 (average duration) ES2 (average duration) ES3 (average duration)	-0.71 -0.75 -0.82
Arthur et al. (5)	ES1 (total duration) ES2 (total duration)	-0.25 -0.060
Drai-Zerbib & Baccino (20)	ES1 (average First-Fixation duration) ES2 (average First-Fixation duration) ES3 (average First-Fixation duration) ES4 (average First-Fixation duration)	-1.24 -0.59 -0.70 -1.20
**Subset 2: Number of fixations (count or rate)**		
Goolsby (35)	ES1 (total # of progressive fixations) ES2 (total # of regressive fixations)	0.38 0.45
Waters et al. (104)^4^	ES1 (average # of fixations) ES2 (average # of fixations) ES3 (average # of fixations)	-0.79 -2.12 -1.33
Waters & Underwood (103)	ES1 (average # of fixations)	1.31
Drai-Zerbib & Baccino (18)	ES1 (total # of fixations) ES2 (total # of fixations)	-8.34 (o) -9.27 (o)
Drai-Zerbib & Baccino (19)	ES1 (average # of fixations) ES2 (average # of fixations)	-2.96 -3.35
Arthur et al. (5)	ES1 (total # of fixations) ES2 (total # of fixations)	0.39 0.53
Lörch (58)	ES1 (total # of fixations)	-0.14
**Subset 3: Saccade amplitude (in pixels or cm)**		
Goolsby (35)	ES1 (average amplitude on progressive saccades) ES2 (average amplitude on regressive saccades)	-0.07 -0.44
Waters & Underwood (103)	ES1 (average amplitude on progressive saccades)	0.26
Drai-Zerbib & Baccino (18)	ES1 (average amplitude on progressive saccades) ES2 (average amplitude on regressive saccades)	0.056 0.11
Penttinen et al. (70)	ES1 (average amplitude) ES2 (average amplitude) ES3 (average amplitude)	-0.08 0.27 0.36
**Subset 4: Gaze duration (in ms)**		
Waters, Underwood, & Findlay (104)	ES1 (average duration) ES2 (average duration) ES3 (average duration)	-0.84 -2.50 -1.70
Drai-Zerbib & Baccino (20)	ES1 (total duration) ES2 (total duration) ES3 (total duration) ES4 (total duration)	-1.09 -0.48 -1.76 -1.75
Silva & Castro (89)	ES1 (total duration) ES2 (total duration) ES3 (total duration) ES4 (total duration)	-2.30 -0.10 1.47 -1.82

*Note.* The number given after each ES was attributed when multiple effect sizes were computed from one single study and with the same participants. A negative ES indicates that experts had shorter fixation durations, a smaller number of fixations, a shorter saccade amplitude or a shorter gaze duration than non-experts. Analogously, a positive ES indicates that experts had longer fixation durations, a higher number of fixations, a longer saccade amplitude, and a longer gaze duration than non-experts. (o): outlier effect size

### General study characteristics

Across all references, it is worth noting that age, level of education, and number of years of musical practice were not systematically reported for each group of participants. Fortunately, the main inclusion criteria used to assign participants to groups depending on their musical expertise were reported.

Overall, it is interesting to note the diversity of eye-tracking devices as well as their main features (sampling rate and accuracy). Both panels of Table 2 summarize the methodological descriptive characteristics, which were extracted from each included study (some of them were used in the moderator analyses). It might have been interesting to consider which algorithm was used to detect fixations and saccades in each study, however, we did not integrate this information in Table 2 because only Drai-Zerbib and Baccino (19) and Penttinen et al. (70) reported it in their study. In the Drai-Zerbib and Baccino (19) study, saccades were determined following a velocity-based algorithm, whereas Penttinen et al. (70) used an algorithm that defined a fixation as each time the gaze was located in a 50 pixels radius during at least 60 ms. Furthermore, Table 3 summarizes effect sizes (Hedges’ *g*) computed for each comparison of each subset (i.e., expert versus non-expert musicians). Overall, the included references concerned a total of 512 participants (with several comparisons involving the same participants).

### Effect size analyses

#### Subset 1: Fixation duration

Weighted mean effect size – Primary analyses were conducted on 29 effect size estimates from 10 different studies. The overall weighted mean effect size across all estimates was *g* = -1.42 (95% CI [-2.96, 0.12], *p* = .066) with a between-study standard error of 0.68. Furthermore, heterogeneity was substantial (Higgins’ *I²* = 90.18%). The huge confidence interval and large standard deviation, as well as the high degree of heterogeneity and asymmetrical funnel plot, suggested the presence of outliers with extreme values in this subset.

To remove such extreme values from the analyses, we used two methods of outlier exclusion. The first being the technique described by Harrer et al. (40). With this method, effect sizes are defined as outliers when their 95% confidence interval lies outside the 95% confidence interval of the pooled effect (3; 95; 111). This method enabled us to identify seven effect sizes from two different studies (three out of four effect sizes from Drai-Zerbib & Baccino, 21; the four effect sizes from Drai-Zerbib & Baccino, 19) as outliers.

The second method used to exclude outliers is the one described by Delgado et al. (16). To check the normality assumption, an examination of the Q-Q normal plot, a Kolmogorov-Smirnov test with Lilliefors correction and a chi-squared test were performed. The Kolmogorov-Smirnov (*d* = 0.27, *p* < .05), the Lilliefors correction (*p* < .01) and the chi-squared test (*p* < .001) all indicated an anormal distribution of effect sizes. With this procedure, we were able to identify five effect sizes as outliers: the effect sizes of the Drai-Zerbib and Baccino (19) study and the ES3 of Drai-Zerbib et al. (21) study. After excluding these 5 outliers, normality assumption tests were no longer significant (*d* = 0.13; *p* = n.s; Lilliefors correction, *p* = n.s; *X*^2^ = 1.88; *p* = .17) indicating a normal distribution of the 24 remaining effect sizes. To be as inclusive as possible, we decided to exclude the five effect sizes identified as outliers with the latter method rather than the seven outliers identified with the first one.

We conducted secondary analyses without these five outlier values. The overall weighted mean effect size across all 24 estimates was medium and significant (*g* = -0.72, 95% *CI* [-1.15, -0.30], *p* < .01), with an estimated between-study standard error of 0.18 (Table 4). The significance code used in a Robust Variance Estimation using the R library robumeta is: < .01 *** < .05 ** < .10 * (25). Higgins test suggested no heterogeneity (I^2^ = 0%). A sensitivity test showed that varying the level of correlations for the dependent effects (from *ρ* = 0 to *ρ* = 1) had no impact on *g* and on the estimated between-study variance (*T²*; see Appendix B).

**Figure 2.**
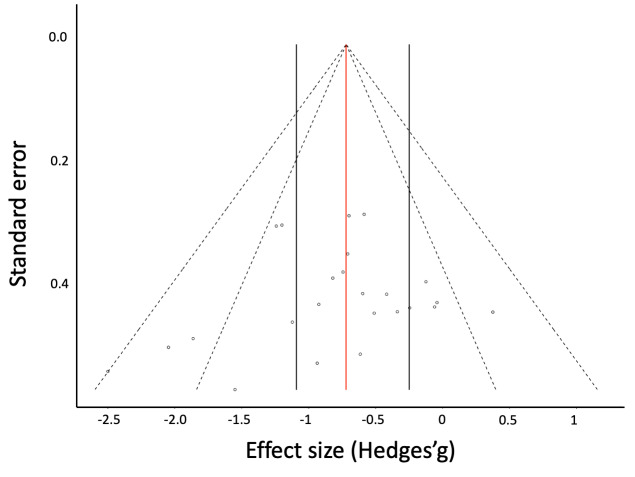
Funnel plot for Subset 1. Each point represents the effect size of one included comparison. The X axis represents Hedges’ g for each comparison, and the Y axis is the corresponding standard error. Red solid line: mean effect size; black solid lines: CI for mean effect size; dashed lines: lower and upper limit values for the 95% CI and 99% CI regions.

Publication bias analysis – After removing outlier values, the nonsignificant Egger’s regression test confirmed that the funnel plot was symmetrical (*z* = -0.96, *p* =.34, Figure 2).

**Table 4.** Summary of the weighted mean effect sizes for each subset (after removing outliers for Subset 1 and Subset 2).

**Subset**	***k***	***g***	***SE***	***df***	***p***	***95% CI***
Subset 1: Fixation duration	24	-0.72	0.18	7.75	.004***	[-1.15, -0.30]
Subset 2: Number of fixations	11	-0.42	0.65	4.98	.548	[-2.10, 1.26]
Subset 3: Saccade amplitude	8	0.061	0.10	2.87	.594	[-0.27, 0.39]
Subset 4: Total gaze duration	11	-1.20	0.28	1.99	.049	[-2.39, -0.008]

*Note.* Weighted mean effect size in terms of Hedges’ *g*; k: number of effect sizes; SE: between-study standard error; *df:* adjusted degrees of freedom; *CI*: confidence interval. Results are not reliable when *df* < 4. Significance code: < .01***.

#### Subset 2: Number of fixations

Weighted mean effect size – Primary analyses were conducted on 13 effect size estimates from seven different studies. The overall weighted mean effect size across all 13 effect size estimates was *g* = -1.48 (95% *CI* [-4.45, 1.49], *p* = .27) with an estimated between-study standard error of 1.21. Furthermore, heterogeneity was substantial (Higgins’ *I²* = 92.93%). As for Subset 1, we had to remove extreme values from the analyses. In this way, we identified two effect sizes as outliers; these effect sizes came from Drai-Zerbib and Baccino (18).

**Figure 3.**
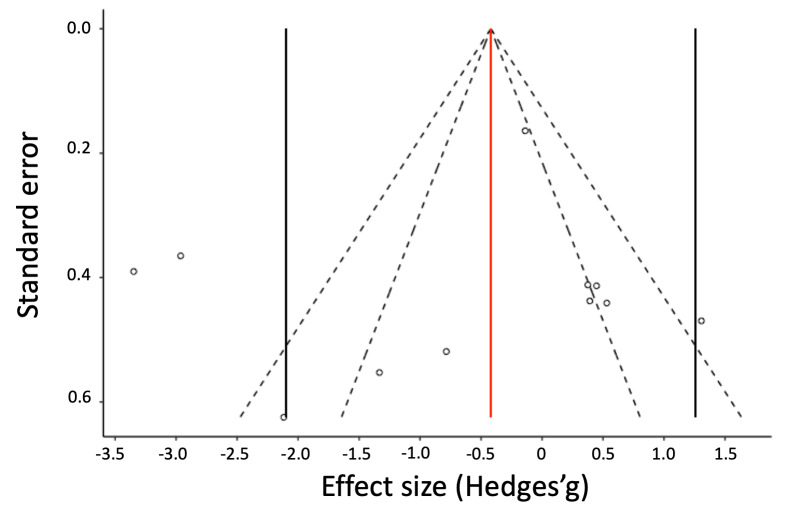
Funnel plot for Subset 2.

Secondary analyses changed the results, with overall heterogeneity decreasing (Higgins’ *I²* = 84.42%) and the funnel plot becoming more symmetrical (Figure 3). Across 11 effect size estimates from six studies, the overall weighted mean effect size was small and not significant (*g* = -0.42, 95% CI [-2.10, 1.26], *p* = .55), with an estimated between-study standard error of 0.65 (Table 4). Finally, varying the assumed within-study effect size correlation (*ρ*) had no impact on *g* and a small impact on the estimated between-study variance (*T²*; see Appendix C).

Publication bias analysis – The nonsignificant Egger’s regression test confirmed that the funnel plot was symmetrical after removing the two outlier effect sizes (*z* = -0.27, *p* = .79, Figure 3).

#### Subset 3: Saccade amplitude - Subset 4: Gaze duration - Moderator analyses

Effect sizes for saccade amplitude, gaze duration and moderator analyses were performed but did not provide any reliable results due to the lack of statistical power.

## Discussion

In the present meta-analysis, the main question was: how does musical expertise modulate eye movements when reading a musical score? We focused on eye-tracking studies in order to conduct four small meta-analyses quantifying the effect sizes of musical expertise on fixation duration (Subset 1), number of fixations (Subset 2), saccade amplitude (Subset 3), and gaze duration (Subset 4). This field of research gathers a small number of studies so far, and we are aware of the limited generalizability of our results. However, the amount of available data to answer our question was sufficient to investigate usual metrics applied in eye-tracking research on music reading. There are valuable results to provide new research directions. Recent doctoral projects have been conducted on this topic (e.g., 44; 58), and for the last two years an annual conference is exclusively devoted to eye tracking in music and helps promoting new findings on this topic (24). Because this field of research is still growing, we also emphasize the need for further music reading and eye-movement research to contribute to the field of music cognition and more generally of expertise. The present meta-analysis thus provides a first cumulative contribution to the field, and a more comprehensive understanding of eye movement characteristics during music reading as a function of musical expertise.

### The effect of musical expertise on eye-movement metrics

Only the results on Subsets 1 and 2 were reliable enough in terms of statistical power, and thus interpretable. More specifically, the analyses on Subset 1 showed a strong and robust effect of musical expertise on fixation duration. Overall, expert musicians have shorter fixations than non-expert musicians in the context of reading music either with or without a playing/singing performance and whatever the type of musical score (21; 18, 19; 78; 103). Both the absence of publication bias and the confidence interval, which did not overlap zero, enhance the reliability of this result and provide information about the consistency of the effect of musical expertise on the fixation duration (102). This highly replicable result favors the long-term working memory theory, which states that experts encode and retrieve relevant information more rapidly than non-experts (23).

Results on Subset 2 were different because we did not find a significant effect of musical expertise on the number of fixations during music reading, although the weighted mean effect size was relatively large. Experts typically produce fewer fixations than non-experts because of their larger perceptual span and their higher ability to chunk the musical information (86). Nevertheless, the present results do not allow us to conclude that musical expertise influences the number of fixations. This result and the huge confidence interval, which includes zero, is explained by the diversity of effect sizes in this subset. As seen in Table 3, some comparisons led to negative estimates, while some led to positive estimates. Because of the huge variability across the comparisons, the degree of heterogeneity was consistent, and we were not able to conduct moderator analyses to gain a more fine-grained understanding of our results. In addition, we cannot conclude on the effect of tempo as a significant moderator of the number of fixations. However, this result must be taken with caution because the low number of studies in this meta-analysis may have obscured the moderating effect of tempo in the Subset 2. Indeed, a recent review (72) underlines that eye movements can be affected by a chosen tempo, the complexity of the score, or the musician’s level of expertise. However, there are methodological conditions that may justify the absence of tempo control and make it useless, such as when participants are not skilled enough to sight read at a given tempo. That is why number of studies usually not impose a tempo on the musicians. Overall, it would be very interesting if future research could measure the effect of tempo control (given tempo and really executed tempo) across expertise levels in sight reading of music.

The results on Subsets 3 and 4 were inconclusive and may be underpowered due to the small number of comparisons. We discuss this issue in the Limitations section. Overall, the present reliable results suggest that the fixation duration is an eye-movement parameter that is less sensitive to variability across studies than the number of fixations. The consistent results on fixation duration confirm that this parameter can be used as a highly reliable marker of musical expertise, and also as a marker of expertise in general: experts produce shorter fixations than non-experts in their domain of expertise (27). This is less clearly the case regarding the number of fixations because this eye-movement parameter seems to be highly modulated by methodological factors. It would be interesting to distinguish the number of fixations on relevant versus irrelevant information in the musical stimuli, or on complex versus easier areas of the scores as seen in other studies on the effect of expertise reflected by eye movements (27; 86).

The second aim of the present meta-analaysis was to investigate how methodological factors might account for the differences in the eye-movements of expert and non-expert musicians (i.e., the type of reading task, the type of musical stimuli, the criteria used to assess the level of expertise, and the type of dependent eye-tracking variables used to investigate the effect of musical expertise). The main objective was to attempt to explain the less consistent results found in the literature, especially in the subsets in which heterogeneity was detected. The fact that the RVE method detected no heterogeneity in Subset 1 suggests that there was a very low level of interstudy variability due to methodological aspects. However, there was a non-negligible variability in the types of methods used to measure fixation duration (Table 2). By contrast, we assessed high level of interstudy variability in Subset 2. There is considerable diversity in the values of the effect sizes, with some of the comparisons showing positive effect sizes and the others showing negative effect sizes (Table 3).

Overall, none of the moderators contributed to explaining the heterogeneity. Since there were very few degrees of freedom (*df* < 4) the results were not reliable enough to warrant any conclusions. It is uncertain whether the lack of significance indicates a true lack of difference or insufficient power to detect an effect.

### Connecting musical expertise to theories of visual expertise

Because music reading is a multimodal activity, the aim of which is to perform, we expect that some eye-movement behaviors are only domain-specific in some contexts or on the contrary similar to those found in other domains of expertise such as in chess, sports, or medicine. Our mixed results emphasized the need for contrasting a wider variety of eye-movement measures and tasks in music-reading studies to develop new theoretical frameworks that would generalize to the visual expertise literature. Because stimuli are domain-specific, training would lead to a specialized information-processing associated with each domain of expertise (9). Studying music reading through a theoretical perspective would help find the commonalities between domains of expertise. 

In their systematic review on visual expertise across domains (mainly sports and medicine) and visual tasks, Brams et al. (9) categorized the eye-movement metrics into three different processes related to different theories. First, their main results suggested that the visual search rate differed between experts and non-experts (i.e., average fixation duration, average number of fixations, and average number of locations fixated), but the direction of this difference was inconsistent across studies. This is in line with the high level of heterogeneity in our Subset 2 (i.e., number of fixations) with negative and positive effect sizes, suggesting that expert musicians may switch between more or less fixations than non-experts. This could be explained by the fact that expert musicians adapt their visual search rate according to the number of elements that require processing, as found in other domains of expertise (11; 101). Moreover, and related to this point, the results of Brams et al. (9) also support that assessing attention allocation on stimuli was relevant to contrast visual strategies of experts versus non-experts by using musical features. It is likely that guided by their domain-specific knowledge, experts have a higher ability to move their focus from one AOI to another and thus tend to make more fixations of longer durations on relevant AOIs versus less relevant AOIs. These findings support the Information-Reduction Theory (39), notably in tasks where experts deal with complex stimuli, as this is the case in music reading. However, our moderator analyses did not allow us to verify such hypothesis.

Finally, Brams et al.’s results (9) partly support the hypothesis that experts have a greater visual span that allows them to use the parafoveal vision, especially in medicine (i.e., shorter time to first fixation on AOI and longer saccade amplitude), which is in line with the hypothesis of the holistic model of image processing (53). Studies addressing parafoveal information processing in expert musicians also converged to these results as shown by longer saccade amplitudes (88). A larger visual span is essential for a global versus local search, this is particularly appropriate in congruency and note detection tasks during music reading (e.g., 5; 19).

### Limitations

Obviously, our conclusions are limited by several factors. In this domain of research, the number of published studies is limited. The most obvious drawback is that the analysis may be biased by the selection of publications showing positive effects. However, we evaluated that possibility, and we found no publication bias. Second, in Subset 2, there was significant heterogeneity between studies which our moderator analyses failed to explain. It is possible that we failed to capture other methodological parameters, which might modulate the effect of musical expertise. Furthermore, tests of moderators using categorical models can have low statistical power. The consequence is that we cannot be sure whether the lack of significance of a given effect is due to the genuine absence of that effect or to a lack of power (43). When power is low, we should not conclude that there is no relationship between the moderator and variation among effect sizes. We can only conclude that more studies are necessary in order to enhance reliability (40).

Regarding the other types of eye-movement measures, the subsets were far too small to conduct proper analyses. Finally, another issue on which we had no control about was the absence of reported Cohen’s *d* effect sizes in the included studies (94). We had to infer this essential measure based on available information, even sometimes from graphical descriptive data. Such approximates preprocessing analyses might have hindered the quality of the final analyses on weighted mean effect sizes. More systematic report of reliable effect sizes would have allowed for including more individual effects sizes (for the record, we had to exclude 23 comparisons because of missing necessary statistical information) which in return would have strengthened our conclusion.

### Recommendations for further directions

The present meta-analysis highlights the need to conduct systematic and quantitative reviews to validate and quantify consistent results (i.e., on fixation duration) and to explain some inconsistencies in the literature (i.e., concerning the number of fixations). Even though the amount of effect sizes is limited, providing a first cumulative systematic review help shed light on the diversity of studies as well as to propose recommendations for research using eye tracking in music. Related to the limitations listed above, we hope the present results will also help in the formulation of interesting new research questions for the growing community.

First, we identify a need for more explicit definitions of the eye-movement variables, which are collected in music-reading experiments, and this advice might be generalized to a broader community investigating visual expertise. It would be useful to establish a glossary including the labels, definitions but also the relevance of using certain variables depending on the mechanisms, which are studied.

Secondly, the use of AOIs in eye-movement analyses should be given a more prominent place in music-reading experiments instead of only reporting the global results for the musical stimuli taken as a whole. The use of such AOIs should be linked to precise hypotheses (i.e., where and why) and might make it possible to extract relevant eye-movement parameters associated with these hypotheses. Furthermore, researchers have reported findings based on the means of first- and second-pass fixations (e.g., 21), first fixations on a target (20), or average fixation durations (e.g., 71). In 1998, Rayner stressed the importance of comparing first- and second-pass fixations or dwell time inside a specific AOI in addition to average fixation duration without segmentation of the musical stimuli. The observation to the transitions between AOIs would help researchers explore gaze strategies and enable them to report the gaze duration and proportion of the gaze duration devoted to the different AOIs as a proportion of the total gaze duration (37). The use of more refined measures should make it possible to differentiate early versus late visual processing of the musical stimuli.

Third, we believe that music-reading expertise should be explored using a greater variety of eye-movement parameters. More specifically, the frequency of short versus long fixation durations may make it possible to determine whether short or long fixations dominate in expert versus non-expert musicians. Dwell time may provide information on how early during processing different parts of the image are looked at, and this is closely linked to the presence of AOIs in the material. In addition, when comparing the number of fixations between two groups with different levels of musical expertise, it should be relevant to have equal time spent on reading the music. If this is not possible because it is not relevant to do so, then the indication of the fixation rate should be more informative and accurate. More generally, until now, data analysis from eye tracking studies in music expertise has largely focused on synchronic indicators (when an event occurs at a specific point in time) such as duration or saccades rather than diachronic indicators (when an event is considered over time) such as scanpath or transition matrix. The evaluation of the difference or similarity between scanpaths across levels of expertise can provide gaze trajectories through musical partitions, thus reflecting visual exploration profiles (57). To go beyond simple quantification of fixations and saccades, it is also possible to analyze the fixation location as well as the direction of the saccades in order to explore the spatial eye-movement trajectory of musicians on the musical stimuli (e.g., to distinguish between reading and scanning; 88). Moreover, dynamic eye-movement measures (i.e., direction, amplitude, velocity) are interesting parameters to consider, in particular because they are highly dependent on the type of stimuli. For example, the present meta-analysis emphasizes the need for a greater consideration of using the saccade amplitude as a relevant parameter to study the perceptual span in music reading and thus would contribute to enrich the theoretical accounts for musical expertise.

Fourth, the computation of the fixation duration and saccades amplitude depends on the event detection algorithm (i.e., dispersion or velocity-based), related to the sampling rate and implemented in the eye-tracking device. The literature should take into account the diversity of algorithms and report the implemented algorithms in the methods section. However, only Drai-Zerbib and Baccino (19) and Penttinen et al. (70) indicated this information in their study. It is conceivable that the way in which the eye-tracker determines what characterizes the duration and location of a fixation, the amplitude of a saccade or even the way in which blinks are taken into account could be significant low-level technical details impacting the interpretations of eye movements in music reading. For example, it would be interesting to investigate how the fact that a fixation is located on the same point before and after a blink affects the number and duration of fixations. It also appears necessary to distinguish between progressive fixations and regressive fixations in order to reflect different aspects of processing during music reading.

Finally, the fact that we cannot draw conclusions about the effect of musical expertise based on the number of fixations and that we were able to explain the high level of heterogeneity underlines the need for new experiments. For example, a crossed design with expertise and type of task as factors would provide more evidence on the potential task-dependent characteristic of the number of fixation parameters. Related to this point, the review by Sheridan et al. (88) discusses examples of interactions between expertise and complexity in music-reading domain (i.e., they distinguish visual complexity manipulations, notational complexity manipulations, and technical complexity manipulations). To go further, it would be interesting to use these characteristics of the stimulus complexity to contrast the eye-movements of expert versus non-expert musicians.

Overall, these recommendations would help future researchers to investigate a broader range of eye-movement behaviors that will account for all possible hypotheses to explain expertise in music reading, and more generally visual expertise. We also expect that the present work would provide insights for more application-oriented research to understand to what extent eye-movement measures might predict the level of musical expertise and how they could be trained to improve playing performance.

### Ethics and Conflict of Interest

The authors declare that the consents of the article are in agreement with the ethics described in https://biblio.unibe.ch/portal/elibrary/BOP/jemr/ethics.html and that there is no conflict of interest regarding the publication of this paper.

### Acknowledgement

This work was supported by the French Agence Nationale de la Recherche (ANR JCJC MUREA Project, grant ANR-18-CE38-0006-0).

## Footnotes

Details for effect size calculations are available in the Appendices section (Appendix A).

This study included three groups of participants. High-level vs low-level musician comparisons led to ES1, high-level vs novice comparisons led to ES2, and low-level vs novice comparisons led to ES3.

This study included three groups of participants. Less-experienced musician vs novice comparisons led to ES1, more experienced musician vs novice comparisons led to ES2, and more experienced vs less-experienced musician comparisons led to ES3.

This study included three groups of participants. High-level vs low-level musician comparisons led to ES1, high-level vs novice comparisons led to ES2, and low-level vs novice comparisons led to ES3.

## Appendix

### Appendix A. Effect size calculations.

Each effect size indicates the standardized difference in eye movements (for each of the eye-tracking variables) during the music-reading task between expert and non-expert musicians. When effect sizes were not directly provided in the *Results* section of the studies, we used available data to calculate each effect size as well as the standard error of the effect size in accordance with the following formulas:

1. 1) When only standard error *se* was available, standard deviation *sd* was calculated as*:*

$$sd=se*\sqrt{n}$$

1. 2) Cohen’ s *d* was computed as:

$$d=\frac{M1-M2}{S}$$

where *M* is the mean for a given condition and *S* the pooled standard deviation for a between-subjects design, such that:

$$S=\sqrt{\frac{\left(n1-1\right){\left(s1\right)}^{2}+\left(n2-1\right){\left(s2\right)}^{2}}{n1+n2-2}}$$

When only appropriate *F*-values were reported, they were first converted to equivalent *t-*values (14; 82). In the case of studies with independent samples that did not report sufficient information for us to use, *t*-values were used to calculate *d* as follows:

$$d=t\sqrt{\frac{n1+n2}{n1*n2}}$$

(from Rowland’s methods section, 84).

1. 3) The standard error of the effect size for a between-subjects design was computed as:

$$d.se=\sqrt{\frac{n1+n2}{n1*n2}+\frac{d^{2}}{2(n1+n2)}}$$

where *n* is the sample size for a condition, *s* is the standard deviation for a condition, and *se* is the standard error for a condition.

1. 4) For small samples, Cohen’s *d* might produce an overestimate of true effect size. Thus, we calculated Hedges’s *g* for each of the included effect sizes in order to correct for this bias. The following formula was used (41; 42):

$$g=d(1-\frac{3}{4N-9}$$

Where *N* is the total number of participants for within-subject as well as between-subjects designs.

### Appendix B. Sensitivity analyses for the fixation duration

These consist in varying the assumed within-study effect size correlation (*ρ*) and observing the impact on the mean effect size (Hedges’ *g*) as well as on the estimated between study-variance (*T²*).

**Table:**

**Subset 1**	0	.2	.4	.6	.8	1
Mean effect size	-0.722	-0.722	-0.722	-0.722	-0.723	-0.723
*SE*	0.181	0.181	0.181	0.181	0.181	0.181
*T^2^*	0.000	0.000	0.006	0.012	0.019	0.025

### Appendix C. Sensitivity analyses for the number of fixations

**Table:**

**Subset 2**	0	.2	.4	.6	.8	1
Mean effect size	-0.421	-0.421	-0.421	-0.421	-0.421	-0.421
*SE*	0.653	0.653	0.653	0.653	0.653	0.653
*T^2^*	1.981	1.985	1.989	1.993	1.997	2.001
